# Bacterial Profile and Antibiotic Susceptibility Pattern of Urinary Tract Infection among Pregnant Women Attending Antenatal Care at a Tertiary Care Hospital in Southern Ethiopia

**DOI:** 10.1155/2020/5321276

**Published:** 2020-12-24

**Authors:** Ashenafi Tula, Abraham Mikru, Tsegaye Alemayehu, Beyene Dobo

**Affiliations:** ^1^Hawassa University College of Computational and Natural Science Department of Biology, Awassa, Ethiopia; ^2^Hawassa University College of Medicine and Health Science School of Medical Laboratory, Awassa, Ethiopia

## Abstract

**Background:**

Urinary tract infection is one of the most common bacterial infections encountered in pregnant women with significant morbidity. This study aimed to determine the bacterial profile and its antimicrobial susceptibility pattern of urinary tract infection among pregnant women attending antenatal care (ANC) at a Hawassa University Comprehensive Specialized Hospital (HUCSH), Southern Ethiopia.

**Method:**

A cross-sectional study was conducted in which consecutive pregnant women enrolled in the study from March to June 2019. The structured questionnaire used to collect sociodemographic and clinical data in a face-to-face interview. Midstream urine was collected from pregnant women using sterile containers. Culture and sensitivity were performed using a standard operating procedure of the microbiology laboratory. Data entry and analysis were conducted using the statistical package for social sciences (SPSS) version 20. Descriptive and logistic regression was used to conduct the output of the data. The odds ratio at 95% confidence interval was considered as a statistically significant association with a *p* value <0.05.

**Result:**

The overall magnitude of urinary tract infection in this study was 7.8% (4.7–10.8%). *Escherichia coli* was found to be the most frequently isolated (47.8%), followed by *Klebsiella pneumoniae* (17.4%), *Staphylococcus aureus* (8.7%), *Klebsiella ozaenae*, *Klebsiella rhinoscleromatis*, *Citrobacter* spp., *Salmonella* group A, *Staphylococcus saprophyticus*, and *Enterobacter cloacae* each (4.3%). Gram-negative bacteria were sensitive to 78.3%, 91.3%, and 100% of ciprofloxacin, gentamicin, and nitrofurantoin, respectively. Gram-positive bacteria were sensitive to clindamycin (100%), gentamicin (100%), and nitrofurantoin (100%) and fully resistant to ceftriaxone (100%) and cefuroxime (100%). There is no statistically significant association (*p* < 0.05) between the risk factor of urinary tract infection and UTI.

**Conclusion:**

The overall prevalence of urinary tract infection among pregnant women attending antenatal care was 7.8%. *Escherichia coli* were the dominant isolate followed by *Klebsiella pneumoniae.* Gram-negative isolates are highly sensitive to ciprofloxacin, gentamicin, nitrofurantoin, and ceftriaxone and Gram-positive isolates to gentamicin, clindamycin, and nitrofurantoin. Most of the bacteria are resistant to cotrimoxazole and cefuroxime. There is no statistically significantly associated variable. Screening for the presence of urinary tract infection during pregnancy will improve the quality of antenatal care further reducing complication. The above antibiotics can be prescribed based on the side effect to pregnant women in case empirical treatment is mandatory in the study area.

## 1. Introduction

Urinary tract infection (UTI) is an infection caused by the existence and growth of microbes somewhere in the urinary tract [1]. It is the commonest health problem worldwide, especially in developing countries [2]. It is usually due to bacteria from the digestive tracts, which climbs the opening of the urethra and begin to multiply to cause infection [3, 4]. It is estimated that about 10 to 20% of all women suffer from UTIs at some point in their life associated with significant morbidity for both mother and fetus [5]. It may lead to unfavourable pregnancy outcomes and complications like preterm delivery, low birth weight, pre-eclamptic toxaemia, and anaemia [6].

Urinary tract infection can be either symptomatic or asymptomatic. Symptomatic urinary tract infection (SUTI) is a patient with significant bacteriuria who has symptoms to the UTI, whereas a condition characterized by lack of symptoms of UTI with significant bacterial yielding positive urine cultures (≥10^5^ colony forming units/millilitre (CFU/ML) is called an asymptomatic urinary tract infection (ASUTI) [7].

It can be caused by a variety of bacteria, including *Escherichia coli* (*E. coli*), *Proteus mirabilis* (*P. mirabilis*), *Klebsiella* species*, Pseudomonas aeruginosa* (*P. aeruginosa*), *Enterobacter* species, *Enterococci*, *Gardnerella vaginalis* (*G. vaginalis*), *Ureaplasma urealyticum* (*U. urealyticum*), *Streptococcus agalactiae* (*S. agalactiae*), *Staphylococcus aureus* (*S. aureus*), *Staphylococcus saprophyticus* (*S. saprophyticus*), and *Staphylococcus haemolyticus* (*S. haemolyticus*) [8, 9].

In different studies, the risk factors for UTI in pregnant women are varied. A variety of factors is associated with UTI, which include age, parity, gravidity, pregnancy, and association of illnesses enhancing the situation of the infection [10, 11]. Illiteracy, history of sexual activity, low socioeconomic monthly income, multiparty, and past history of UTI are also reported as significant risk factors for UTI during pregnancy [12, 13].

Management of UTIs is usually empirical in many developing countries, even though antimicrobial resistance among the pathogens that cause UTI is increasing worldwide including Ethiopia [14–20]. This is further complicated by the fact that, in most hospitals, routine culture and sensitivity testing have not been done and treatment is subjective [21]. Therefore, this study aimed to determine the bacterial profile, antibiotic susceptibility pattern, and associated risk factors of UTI among pregnant women attending ANC in HUCSH, Southern Ethiopia.

## 2. Materials and Methods

### 2.1. Study Area

This study was conducted in HUCSH, Sidama Regional State, South of Ethiopia. The Hospital is located at 279 kilometres (Km) from Addis Ababa in the south of Ethiopia. It is a tertiary hospital found in the Sidama region), that gives services for over 3.5 million people and serves as a referral hospital for the neighbouring regional state. The antenatal clinic has an annual attendance of 1000 pregnant women. The hospital has qualified staff members, including midwifery nurses, doctors, and gynaecologists. The city is divided into eight sub-cities with a total population of 371,826 of which 191,352 are male and 180,474 are female. The city is located at 70° 03” latitude and 80° 29” east longitude. It also lies at an altitude of 1708 m above sea level [22].

### 2.2. Study Design

A cross-sectional study was conducted among pregnant women attending ANC at HUCSH from March to June 2019. All pregnant women that attended ANC at HUCSH during the study period were the source population. The study population was selected consecutively for the study. All pregnant women without current antibiotic therapy and willing to participate were included. Pregnant women taking antibiotic treatment for any diseases and that did not volunteer to participate in the study were excluded. The presence of UTI was the dependent variable, whereas the sociodemographic factors (age, sex, marital status, educational level, occupation, and residence) and clinical feature (parity, gravidity, history of catheterization, trimester, history of UTI, presence of diabetes, and chronic renal disease) were the independent variable of the study.

### 2.3. Sample Size Determination and Sampling Technique

Sample size was determined using a single proportion formula taking estimated prevalence 26% from the previous study from Bale Goba (20), the margin of error 5 % at 95% confidence interval. Since the average annual total number of pregnant women who visit the HUCSH for follow-up was approximately 1000 (which is <10,000) from the record book referred, the required maximum sample of 296 was obtained from the above estimate by making some adjustments, a convenient sampling technique in which consecutive patients enrolled in the study until the sample size was achieved.

### 2.4. Data Collection

Trained data collector (nurses) collected sociodemographic and clinical data using a structured questionnaire in a face-to-face interview. The interview was conducted if and only if the study participant volunteered to participate in the study.

### 2.5. Laboratory Analysis

Each selected pregnant woman was instructed on how to collect midstream urine specimen by a trained interviewer. Before collection, they were advised to clean their hands with water and soap, and then cleanse the periurethral area with a sterile cotton swab soaked in normal saline. Accordingly, about 20 ml of urine specimen was collected in a sterile screw-capped, wide-mouth cup labelled with a unique sample number, date, and time of collection. The specimen was immediately delivered to the microbiology laboratory of the HUCSH within one hour of collection for processing. It was stored at 4°C in case delay is mandatory.

### 2.6. Culture and Identification

Blood agar plate (BAP) and MacConkey (MAC) agar (Oxoid Ltd, England) were prepared as per the instruction of the manufacturer used for inoculation of urine specimen. Standard calibrated loop (0.001 ml) was used to inoculate urine specimen BAP and MAC agar. Streaked culture plates were incubated at 37°C for 24 hours and were inspected for bacterial growth on the next day and colonies were counted manually on BAP. Urine cultures, which grew ≥10^5^ (CFUs)/ml of urine sample consisting of one type of colony morphology, were considered as significant bacteriuria. Culture isolated with two or more bacteria was considered as mixed flora that may be due to contamination [5].

The bacteria were identified by colony morphology, Gram staining, odour, and presence of hemolysis on their respective media and confirmed by the pattern of biochemical reactions using standard procedures. Gram-positive bacteria were identified using Gram reaction, catalase, and coagulase test. Gram-negatives rods were identified with a series of biochemical tests such as indole, citrate utilization, oxidase, lactose fermentation, urea hydrolysis, and motility [23, 24].

### 2.7. Antimicrobial Susceptibility Test

Antimicrobial susceptibility testing was performed by disk diffusion method as described by Kirby-Bauer [25] on Mueller-Hinton agar (MHA) (Oxoid Ltd, England) using Clinical and Laboratory Standards Institute [26], [26] commonly prescribed antibiotics. Different antibiotics were used based on the recommendation of 2019 CLSI guideline that includes ciprofloxacin (5 *μ*g), norfloxacin (10 *μ*g), gentamicin (10 *μ*g), chloramphenicol (30 *μ*g), ceftriaxone (30 *μ*g), nitrofurantoin (300 *μ*g), clindamycin (2 *μ*g), ceftazidime (30 *μ*g), and cefuroxime (30 *μ*g). Loopful of pure culture was taken from a colony suspended in 5 ml sterile saline (0.85% NaCl). The turbidity of the suspension was then adjusted to the optical density of 0.5 McFarland standards. A sterile cotton swab was used to inoculate the suspension on MHA and the plates were incubated at 37°C for 24 hours. Then, the diameters of the zone of bacterial growth inhibition around the discs were measured to the nearest millimeter using a metallic calliper and interpreted as sensitive or resistant according to the standardized table provided by CLSI, 2019.

### 2.8. Quality Control

Routine sterility testing of the media was performed by placing 5% of the batch in the incubator at 37°C overnight and checking for the presence of turbidity in a fluid medium and presence of growth in solid media. Standard strains American type culture collection (ATCC) of *E. coli* (ATCC 25922) and *S. aureus* (ATCC 25923) and *P. aeruginosa* (ATCC 27853) was used to check for performance of the media and biochemical tests.

### 2.9. Data Analysis

Sociodemographic, clinical, and laboratory data were entered and analyzed using statistical package for the social sciences (SPSS) version 20. Bivariate and multivariate analyses were performed to evaluate whether individual predictors are associated with UTI. The result of UTI was segmented as the presence or absence of UTI. Urinary tract infections were tested against presumed UTI-associated variables for relationship evaluation. Bivariate analysis was performed on all variables and all variables with a *p* value ≤0.20 were entered in the stepwise forward multivariate logistic regression model. In all cases, *p* value <0.05 was considered as statistically significant at a 95% confidence interval.

### 2.10. Ethical Considerations

The study was approved by the Institutional Review Board [25] of Hawassa University College of Medicine and Health Sciences (HUCMHS). Official permission from HUCSH was obtained. The study was conducted voluntarily after the written consent of each pregnant woman was obtained in an interview before taking a urine sample. Confidentiality of any information related to the patient and clinical history was well kept up. However, the attendant physicians had access to partial medical record based on the responsibility associated with patient treatment.

## 3. Results

### 3.1. Sociodemographic Characteristics

Two hundred ninety-six (296) pregnant women were enrolled in the study with a 100% response rate, of which 124 (41.9%) and 172 (58.1%) study subjects were clinically symptomatic and asymptomatic for UTI, respectively. The majority of the study participants were in the age range of 16–20 years (58.1%). The high mean age rate was 16–20 years (58.1%), whereas the low age rate was ≥26 (4.4%). Most of the 213 (72.0%) were from urban areas, whereas 83 (28.0%) were rural. Among the study participants, 296 (100%) were married in their marital status, elementary school 97 (32.8%) in their educational status, 125 (42.2%) were housewives in their occupation, and their monthly income levels were in the income range of 501–1000, 132 (44.6%) (Table 1).

### 3.2. Clinical Features

Based on their gravidity, 89 (30.1%), 104 (35.1%), 74 (25.0%), and 29 (9.8%) had first gravid, second gravid, third gravid, and above fourth gravid, respectively. Concerning trimester of pregnancy, regarding gestational age, 137 (46.3%), 106 (35.8%), and 53 (17.9%) pregnant women were in the 3^rd^, 2^nd^, and 1^st^ trimester, respectively. On the other hand on their parity, 89 (30.1%), 99 (33.4%), and 108 (36.5%) were nulliparous, primiparous, and multiparous, respectively. About 27 (9.1%), 9 (3.0%), 16 (5.4%), 26 (8.8%), and 46 (15.5%) of the study subjects had a history of UTI, previous history of catheterization, diabetes mellitus, kidney problem, and gynaecological surgery, respectively (Table 1).

### 3.3. Magnitude of UTI

**Figure 1:**
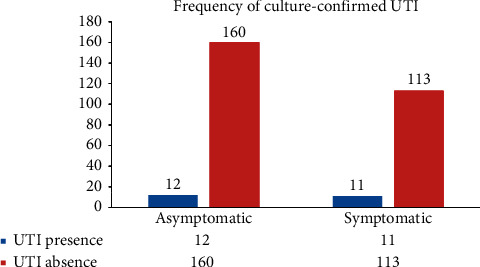
Symptomatic and asymptomatic UTI among pregnant women attending ANC at HUCSH, Southern Ethiopia, from March to June 2019.

The Overall magnitude of UTI in this study was 7.8 (4.7–10.8%). Of the total 296, 172 (58.1%) of pregnant women were clinically suspected as ASUTI, of which 12 (7.0%) had culture-confirmed UTI. The rest (124) (41.9%) were clinically suspected as SUTI, 11 (8.9%) confirmed as UTI in culture (Figure 1).

### 3.4. Bacterial Profile

**Figure 2:**
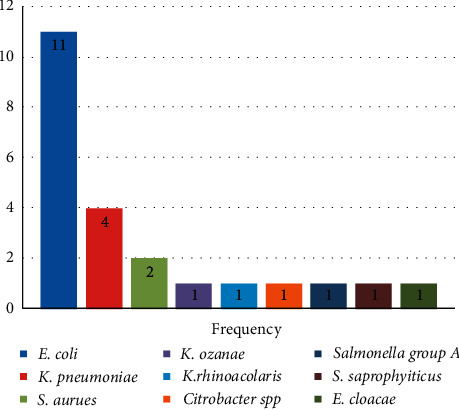
The bacterial profile isolated from pregnant women attending ANC at HUCSH, Southern Ethiopia, from March to June 2019.

Nine different bacteria were isolated in this study. The majority of the isolates were Gram-negative. *E. coli* was found to be the most frequently isolated (11) (47.8%), followed by *K. pneumoniae* (4) (17.4%), *S. aureus* (2) (8.7%), *K. ozaenae* (1) (4.3%), *K. rhinoscleromatis* (1) (4.3%), *Citrobacter* spp. (1) (4.3%), *Salmonella group A* (1) (4.3%), *S. saprophyticus* (1) (4.3%), and *E. cloacae* (1) (4.3%) (Figure 2).

### 3.5. Antimicrobial Susceptibility Pattern

Gram-negative bacteria showed a high level of sensitivity to CIP (78.3%), GEN (91.3%), and NIT (100%) and high resistance against COT (68.2%) and CRX (47.8%). However, they showed relatively low resistance to CIP (21.7%), CTR (26.1%), and CAZ (28.6%). *E. coli* was sensitive to GEN (100%), NIT (100%), CTR (90.9%), NOR (90.9%), CIP (81.8%), CRX (81.8%), and CAZ (72.7%) and resistant to COT (81.8%) and CAZ (27.3%). *K. pneumoniae* was sensitive to GEN (100%), NIT (100%), NOR (75%), CAZ (75%), CIP (75%), and COT (75%). *K. rhinoscleromatis* was fully resistant to CIP (100%), COT (100%), GEN (100%), CTR (100%), NOR (100%), CAZ (100%), and CRX (100%). *Citrobacter* species were fully (100%) resistant to COT, GEN, NOR, and CRX. Gram-positive isolates were highly susceptible to CLD (100%), GEN (100%), and NIT (100%) and fully resistant to CTR (100%) and CRX (100%) (Table 2).

### 3.6. Associated Risk Factors

We conducted binary logistic regression analysis, whether there is an association between UTI and different variables. In bivariate analysis, variables were age range (16–20 years) (COR = .376, 95% CI (.074–1.910), *p*=0.238), being urban residence (COR = 4.430, 95% CI (1.015–19.33), *p*=0.048), their educational status of elementary (COR = .127, 95% CI (.016–1.016), *p*=0.052), being a high school (COR = .474, 95% CI (.174–1.286), *p*=0.143), and completed higher education (COR = .358, 95% CI (.109–1.181), *p*=0.092). In addition to this factor, occupation of being government workers (COR = 4.370, 95% CI (.540–35.395), *p*=0.167), those who had no history of catheterization (COR = 3.619, 95% CI (.707–18.528), *p*=0.123), and those who had multipara parity (COR = .521, 95% CI (.172–1.582), *p*=0.250) were candidate variables for multivariate analysis with *p* value ≤.25 (Table 1).

In multivariate analysis, taking all variables *p* < 0.2, there was no variable statically significant. All variables were *p* value ≥.05 (Table 3).

## 4. Discussion

The overall prevalence of UTI among pregnant women attending ANC at HUCSH was 23 (7.8 (4.7–10.8%)) during the study period. This was comparable to a study reported from India (7.7%) [27], Bahir Dar (9.5%) [28], and Gondar (10.4%) [19], but it was lower than studies conducted in different parts of the world: Addis Ababa (11.6%) [18], Dire Dawa (14%) [29], Sudan (14%) [30], Tanzania (15.5%) [8], Saudi Arabia (20%) [31], Bale Goba (26%) (20), Libya (30%) [32], Niger (75%) [33], and Iraq (64.6%) [34]. The variation might be due to the difference in sample size and geographical location [19].

In this study, 7.0% of the women were with ASUTI, which is lower than studies conducted in Nigeria (28.5%) [35], Pakistan (28%) [36], Bale Goba (22%) [20], Bahir Dar (18.9%) [19], Spain 16%) [37], Sudan (14.7%) [30], Bangladesh (12%) [38], Tanzania (13%) [8], Dire Dawa (11%) [29], Addis Ababa (10.6%) [18], and Gondar (10.2%) [28]. It was comparable with studies performed in Ghana (7.3%) [39], Saudi Arabia (8%) [31], and Iraq (6.3%) [34]. Our study indicated that SUTIs were 8.9%; this was comparable to the findings reported from Bahir Dar (8.5%) [19] and Iraq (8.0%) [34]. Our findings were lower than those from Bale Goba (35.3%) [20], Addis Ababa (20%) [18], Tanzania (17.9%) [8], Dire Dawa (17%) [29], Gondar (15.9%) [28], Sudan (12.1%) [30], and Saudi Arabia (12%) [31].

In this study, Gram-negative bacterial isolates were more prevalent than Gram-positive bacterial isolates (77.8% vs. 22.2%). In agreement with our finding studies from Addis Ababa [18], Gondar [19], India [27], Sudan [30], and Bahir Dar [40] reported similarly as gram negative were predominant bacteria in their finding. The most common uropathogen identified in this study was *E. coli* (47.8%), which is in agreement with studies conducted in Addis Ababa [18], Sudan [30], Bahir Dar [28], and Gondar [19]. *E. coli* is the most common microorganism in the vaginal and rectal area [19]. Anatomical and functional changes and difficulty of maintaining personal hygiene during pregnancy might increase the risk of acquiring a UTI by *E. coli* [41]. The major contributing factor for isolating higher *E. coli* is due to urine stasis in pregnancy, which favours *E. coli* strain colonization [28, 42].

The knowledge of the antimicrobial susceptibility pattern of UTI pathogens was very important for the clinician to select and use the most effective antimicrobial agent for the treatment of a patient with UTI [18]. Our study revealed that CIP (80%), GEN (90%), NIT (100%), CTR (80%), NOR (80%), CAZ (73.7%), and CRX (60%) were relatively effective antibiotics against Gram-negative uropathogens. However, COT (70%), NOR (80%), and CRX (40%) exhibited a high level of resistance by Gram-negative isolates. This finding is in agreement with the study conducted in Gondar [43], Addis Ababa [18], Sudan [30], Nigeria [44], Dire Dawa [29], and Bale Goba [20]. On the other hand, Gram-positive bacteria in the current studies were 100% sensitive to CLD, GEN, and NIT, which is in agreement with the findings of some other studies [18, 45]. In the present study, more than 65% of the Gram-positive bacteria were susceptible to CIP (67.7%), NOR (67.7%), and COT (50%). However, CTR (100%), CAZ (66.7%), and CRX (100%) exhibited a high level of resistance by Gram-positive isolates. In the present study, NIT and GEN were observed as the highest sensitive pattern when the average of susceptibility was commonly considered for both Gram-negative and Gram-positive isolates, which were followed by CIP, CTR, and NOR. The present study showed that 100% of the Gram-positive and 90% of the Gram-negative bacteria were susceptible to GEN, which is in line with previous studies from Ethiopia [43, 46]. In this study, CLD was observed as the highest sensitive one when the average of susceptibility was considered for only Gram-positive isolates, followed by NIT, GEN, and CIP. The low resistance level here in our study may be due to the source of an organism being from the community, not hospital-acquired.

Our study assessed whether there is an association of UTI with sociodemographic and different clinical variables. However, there was no statistically associated variable for UTI among pregnant women attending ANC at HUCSH. This is in agreement with studies in Ethiopia [19, 40] and Sudan [30]. In contrast to this, a finding from somewhere else showed that maternal age and gravidity are risk factors for UTI among pregnant women [47]. Lack of significant association between the possible independent risk factors and prevalence of UTI in the present study could be due to the difference in the methodology, study population (living standards), and sample size in the study [19].

## 5. Conclusions

The overall prevalence of UTI among pregnant women attending at HUCSH is high. *E. coli* was the dominant isolate followed by *K. pneumoniae.* Gram-negative isolates were shown to be highly sensitive to CIP, GEN, NIT, and CTR, whereas Gram-positive isolates were highly sensitive to GEN, CLD, and NIT. Most of the bacterial isolates are resistant to COT and CRX. There is no variable statistically significantly associated with UTI. Screening for the presence of UTI during pregnancy will improve the quality of antenatal care, further reducing complication. The above antibiotics can be prescribed based on the side effect to pregnant women in case empirical treatment is mandatory in the study area.

## Figures and Tables

**Table 1:** Bivariate analysis for the assessment of factors associated with UTI among pregnant women attending ANC at HUCSH, Southern Ethiopia, from March to June 2019.

Variables	Frequency (%)	UTI		COR (95% CI)	*p* value
		Presence (%)	Absence (%)		
Age in years					
16–20	172 (58.1)	11 (6.4)	161 (93.6)	0.376 (0.074–1.910)	0.238
21–25	111 (37.5)	10 (9.0)	101 (91.0)	0.545 (0.106–2.809)	0.468
≥26	13 (4.4)	2 (16.7)	11 (83.3)	1	—

Residence					
Urban	213 (72.0)	21 (9.9)	192 (90.1)	4.430 (1.015–19.33)	0.048
Rural	83 (28.0)	2 (2.4)	81 (97.6)	1	—

Educational status					
Read and Write	49 (16.6)	1 (2.0)	48 (98.0)	1	—
Elementary	97 (32.8)	7 (7.2)	90 (92.8)	0.127 (0.016–1.016)	0.052
High school	72 (24.3)	4 (5.6)	68 (94.4)	0.474 (0.174–1.286)	0.143
Higher education	78 (26.4)	11 (14.1)	67 (85.9)	0.358 (0.109–1.181)	0.092

Occupation					
Housewife	125 (42.2)	6 (5.6)	101 (94.4)	1	—
Merchant	89 (30.1)	11 (13.1)	73 (86.9)	1.723 (0.199–14.892)	0.621
Government employee	48 (16.2)	5 (6.7)	70 (93.3)	4.370 (0.540–35.395)	0.167
Others	34 (11.5)	1 (3.3)	29 (96.7)	2.071 (0.232–18.513)	0.515

Monthly income (Birr)					
≤500	64 (21.6)	3 (6.0)	47 (94.0)	1	—
501–1000	132 (44.6)	8 (7.4)	100 (92.6)	1.253 (0.318–4.939)	0.747
1001–1500	73 (24.7)	6 (11.8)	45 (88.2)	2.089 (0.492–8.860)	0.318
>=1501	27 (9.1)	6 (6.9)	81 (93.1)	1.160 (0.277–4.8)	0.839

Flank pain					
Presence	43 (14.5)	3 (7.0)	40 (93.0)	1	—
Absence	253 (85.5)	20 (7.9)	233 (92.1)	1.144 (0.325–4.031)	0.834

Dysuria					
Presence	32 (10.8)	1 (3.1)	31 (96.9)	1	—
Absence	264 (89.2)	22 (8.3)	242 (91.7)	0.355 (0.046–2.725)	0.319

Urgency of urination					
Presence	59 (19.9)	5 (8.5)	54 (91.5)	1	—
Absence	237 (80.1)	18 (7.6)	219 (92.4)	1.127 (0.400–3.170)	0.821

Suprapubic pain					
Presence	39 (13.2)	4 (10.3)	35 (89.7)	1	—
Absence	257 (86.8)	19 (7.4)	238 (92.6)	1.432 (0.460–4.45)	0.536

Hematuria					
Presence	166 (56.1)	14 (8.4)	152 (91.6)	1.238 (0.518–2.958)	0.630
Absence	130 (43.9)	9 (6.9)	121 (93.1)	1	—

History of UTI					
Presence	27 (9.1)	3 (11.1)	24 (88.9)	1	—
Absence	269 (90.9)	20 (7.4)	249 (92.6)	1.556 (0.431–5.618)	0.500

History of catheterization					
Presence	9 (3.0)	2 (22.2)	7 (77.8)	1	—
Absence	287 (97.0)	21 (7.3)	266 (92.7)	3.619 (0.707–18.528)	0.123

Current symptoms					
Presence	42 (14.2)	3 (7.1)	39 (92.9)	1	—
Absence	254 (85.8)	20 (7.9)	234 (92.1)	900 (0.255–3.173)	0.870

Gravidity					
1^st^	89 (30.1)	8 (9.0)	81 (91.0)	1.333 (0.267–6.667)	0.726
2^nd^	104 (35.1)	6 (5.8)	98 (94.2)	0.827 (0.158–4.330)	0.822
3^rd^	74 (25.0)	7 (9.5)	67 (90.5)	1.410 (0.275–7.226)	0.680
≥4^th^	29 (9.8)	2 (6.9)	27 (93.1)	1	—

Gestational age					
1^st^ trimester	53 (17.9)	4 (7.5)	49 (92.5)	1	—
2^nd^ trimester	106 (35.8)	8 (7.5)	98 (92.5)	0.935 (0.284–3.077)	0.891
3^rd^ trimester	137 (46.3)	11 (8.0)	126 (92.0)	0.935 (0.362–2.413)	0.890

History of diabetes status					
Presence	16 (5.4)	1 (6.2)	15 (93.8)	1	—
Absence	235 (79.4)	18 (7.7)	217 (92.3)	0.683 (0.071–6.612)	0.742
Unknown	45 (15.2)	4 (8.9)	41 (91.1)	0.850 (0.274–2.642)	0.779

Renal problem					
Presence	26 (8.8)	2 (7.7)	24 (92.3)	1	—
Absence	270 (91.2)	21 (7.8)	249 (92.2)	1.012 (0.224–4.580)	0.988

Gynaecological surgery					
Presence	46 (15.5)	2 (4.3)	44 (95.7)	1	—
Absence	250 (84.5)	21 (8.4)	229 (91.6)	2.017 (0.457–8.915)	0.355

Prolonged antibiotic therapy					
Presence	68 (23.0)	5 (7.4)	63 (92.6)	1	—
Absence	228 (77.0)	18 (7.9)	210 (92.1)	1.080 (0.386–3.025)	0.884

Previous use of antibiotics					
Yes	31 (10.5)	1 (3.2)	30 (96.8)	1	—
No	265 (89.5)	22 (8.3)	243 (91.7)	2.716 (0.353–20.880)	0.337

Parity					
Nulliparous	89 (30.1)	8 (9.0)	81 (91.0)	1	—
Primiparous	99 (33.4)	5 (5.1)	94 (94.9)	0.968 (0.365–2.566)	0.948
Multipara	108 (36.5)	10 (9.3)	98 (90.7)	0.521 (0.172–1.582)	0.250

**Table 2:** Antimicrobial susceptibility pattern of gram-negative and positive bacteria isolated from urine culture of pregnant women attending ANC at HUCSH (as shown in Table 1) (*n* = 23).

Isolates (no.)	AST pattern	Antimicrobial agents tested								
		CIP	COT	GEN	NIT	CTR	NOR	CAZ	CRX	CLD
*E. coli* (11)	S	9	2	11	11	10	10	8	9	—
	R	2	9	0	0	1	1	3	2	—

*K. pneumoniae* (4)	S	3	3	4	4	2	3	3	2	—
	R	1	1	0	0	2	1	1	2	—

*K. rhinoscleromatis* (1)	S	0	0	0	1	0	0	0	0	—
	R	1	1	1	0	1	1	1	1	—

*Citrobacter* spp. (1)	S	1	0	0	1	1	0		0	—
	R	0	1	1	0	0	1		1	—

*K. ozaenae* (1)	S	1	1	1	1	1	1	1	0	—
	R	0	0	0	0	0	0	0	1	—

*Salmonella Group A* (1)	S	1	0	1	1	1	1	1	1	—
	R	0	1	0	0	0	0	0	0	—

*E. cloaca* (1)	S	1	0	1	1	1	1	1	0	—
	R	0	1	0	0	0	0	0	1	—

**Subtotal (20)**	**S (%)**	**16(80)**	**6(30)**	**18(90)**	**20(100)**	**16(80)**	**16(80)**	**14(73.7)**	**12(60)**	—
	**R (%)**	**4(20)**	**14(70)**	**2(10)**	**0(0)**	**4(20)**	**4(20)**	**5(26.3)**	**8(40)**	—

*S. aureus* (2)	S	1	1	2	2	0	1	1	0	2
	R	1	1	0	0	2	1	1	2	0

*S. saprophyticus* (1)	S	1	—	1	1	0	1	0	0	1
	R	0	—	0	0	1	0	1	1	0

**Subtotal (3)**	**S (%)**	**2(66.7)**	**1(50)**	**3(100)**	**3(100)**	**0(0)**	**2(66.7)**	**1(33.3)**	**0(0)**	**3(100)**
	**R (%)**	**1(33.3)**	**1(50)**	**0(0)**	**0(0)**	**3(100)**	**1(33.3)**	**2(66.6)**	**3(100)**	**0(0)**

S: sensitive; R: resistance; CIP: ciprofloxacin; COT: cotrimoxazole; GEN: gentamicin; NIT: nitrofurantoin; CTR: ceftriaxone; NOR: norfloxacin; CAZ: ceftazidime; CRX: cefuroxime; CLD: clindamycin.

**Table 3:** Multivariate analysis for the assessment of factors associated with UTI among pregnant women attending ANC at HUCSH, Southern Ethiopia, from March to June 2019.

Variables	Frequency (%)	UTI		COR (95% CI)	*p* value	AOR (95%CI)	*p* value
		Presence (%)	Absence (%)				
Age in years							
16–20	172 (58.1)	11 (6.4)	161 (93.6)	0.376 (0.074–1.910)	0.238	0.226 (0.031–1.633)	0.141
21–25	111 (37.5)	10 (9.0)	101 (91.0)	0.545 (0.106–2.809)	0.468	0.315 (0.047–2.138)	0.237
≥26	13 (4.4)	2 (16.7)	11 (83.3)	1	—	—	—

Residence							
Urban	213 (72.0)	21 (9.9)	192 (90.1)	4.430 (1.015–19.33)	0.048	4.315 (0.939–19.839)	0.060
Rural	83 (28.0)	2 (2.4)	81 (97.6)	1	—	—	—

Educational status							
Read and Write	49 (16.6)	1 (2.0)	48 (98.0)	1	—	—	—
Elementary	97 (32.8)	7 (7.2)	90 (92.8)	0.127 (0.016–1.016)	0.052	3.139 (0.306–32.147)	0.335
High school	72 (24.3)	4 (5.6)	68 (94.4)	0.474 (0.174–1.286)	0.143	2.613 (0.232–29.464)	0.437
Higher education	78 (26.4)	11 (14.1)	67 (85.9)	0.358 (0.109–1.181)	0.092	8.914 (0.926–85.797)	0.058

Occupation							
Housewife	125 (42.2)	6 (5.6)	101 (94.4)	1	—	—	—
Merchant	89 (30.1)	11 (13.1)	73 (86.9)	1.723 (0.199–14.892)	0.621	3.203 (0.992–10.339)	0.052
Government employee	48 (16.2)	5 (6.7)	70 (93.3)	4.370 (0.540–35.395)	0.167	3.418 (0.845–13.828)	0.085
Others	34 (11.5)	1 (3.3)	29 (96.7)	2.071 (0.232–18.513)	0.515	0.538 (0.057–5.121)	0.590

History of catheterization							
Presence	9 (3.0)	2 (22.2)	7 (77.8)	1	—	—	—
Absence	287 (97.0)	21 (7.3)	266 (92.7)	3.619 (0.707–18.528)	0.123	0.173 (0.026–1.140)	0.068

Parity							
Nulliparous	89 (30.1)	8 (9.0)	81 (91.0)	1	—	—	—
Primiparous	99 (33.4)	5 (5.1)	94 (94.9)	0.968 (0.365–2.566)	0.948	0.573 (0.159–2.070)	0.396
Multipara	108 (36.5)	10 (9.3)	98 (90.7)	0.521 (0.172–1.582)	0.250	0.916 (0.267–3.151)	.890

## Data Availability

The data used to support the findings of the study are available from the corresponding author upon request.

## Abbreviations

ANC: Attending antenatal care
ASUTI: Asymptomatic urinary tract infection
ATCC: American type culture collection
CFU: Colony forming units
CLSI: Clinical and Laboratory Standards Institute
HUCSH: Hawassa University Comprehensive Specialized Hospital
NCCLS: National Committee for Clinical Laboratory Standards
SNNPR: Southern Nation Nationality Peoples Region
SUTI: Symptomatic urinary tract infection
UTI: Urinary tract infection.
